# A Case Report of a Wenckebach Phenomenon Occurring during a His-Bundle Pacing Procedure: Is It Atrioventricular Node Pacing?

**DOI:** 10.3390/jcdd9070231

**Published:** 2022-07-19

**Authors:** Chengming Ma, Wenwen Li, Yongmei Cha, Yunlong Xia, Lianjun Gao, Yingxue Dong

**Affiliations:** 1 Department of Cardiology, The First Affiliated Hospital of Dalian Medical University, Dalian 116011, China; dy.mcm@aol.com (C.M.); yunlong_xia@126.com (Y.X.); lianjun_gaophd@163.com (L.G.); 2 Department of Intensive Care Medicine, The First Affiliated Hospital of Dalian Medical University, Dalian 116011, China; wen0303yx@163.com; 3 Department of Cardiovascular Diseases, Mayo Clinic, 200 First Street SW, Rochester, MN 55421, USA; cha.yongmei@mayo.edu

**Keywords:** Wenckebach phenomenon, His-bundle pacing, atrioventricular node

## Abstract

A 70-year-old man with severe valvular cardiomyopathy, permanent atrial fibrillation (AF) with a slow ventricular response, and transient atrioventricular (AV) block, was admitted to our center for severe heart failure and recurrent presyncope. While hospitalized, the coronary computed tomography angiography (CTA) showed huge atriums. We tried His bundle pacing (HBP). HB potential was observed at site A, and the His-ventricular (HV) interval was 68 ms. The duration from the stimulus signal to the onset of paced QRS (S-QRSonset) at site A was 232 ms when pacing at 60 beats per minute (BPM) with the pacing threshold of 2.0 V/0.5 ms. The S-QRSonset was longer than the HV interval and had a notable and progressive prolongation from 252 ms to 456 ms during the pacing at 90 BPM. Then, we pushed another lead a little forward, and the S-QRSonset shortened back to 68 ms, and the paced QRS morphology was the same as the intrinsic QRS morphology with the pacing threshold of 1.5 V/0.5 ms. The progressively prolonged S-QRSonset demonstrated a Wenckebach phenomenon (WP), a well-known electrophysiological characteristic of the AV node (AVN). It is the first time to report an intraoperative AVN-pacing related-WP in a patient with persistent AF. The enlarged atrium might be convenient for capturing the AVN. There are some other potential explanations for this phenomenon. The diameters of atriums decreased significantly, and the symptoms improved after the procedure. This is the first reported case in which we might achieve AVN capture in a patient with persistent AF. Although we ultimately chose HBP for better long-term pacing thresholds, the result of this case suggested that AVN pacing may be possible.

## 1. Introduction

The Wenckebach phenomenon (WP) is a well-known electrophysiological characteristic of the atrioventricular node (AVN). However, its physiological properties are not well understood. There are multiple structures within and around the AVN, including transitional tissue, the inferior nodal extension, the penetrating bundle, the His bundle, atrial and ventricular muscle, the tendon of Todaro, and valves [1,2]. AVN pacing during permanent pacemaker implantation is rare. We herein report the first case of the Wenckebach phenomenon occurring in a heart failure (HF) patient with chronic atrial fibrillation (AF) for more than 30 years during a His-bundle pacing (HBP) procedure. The patient’s symptoms of HF improved significantly after the procedure.

## 2. Case Report

**Figure 1:**
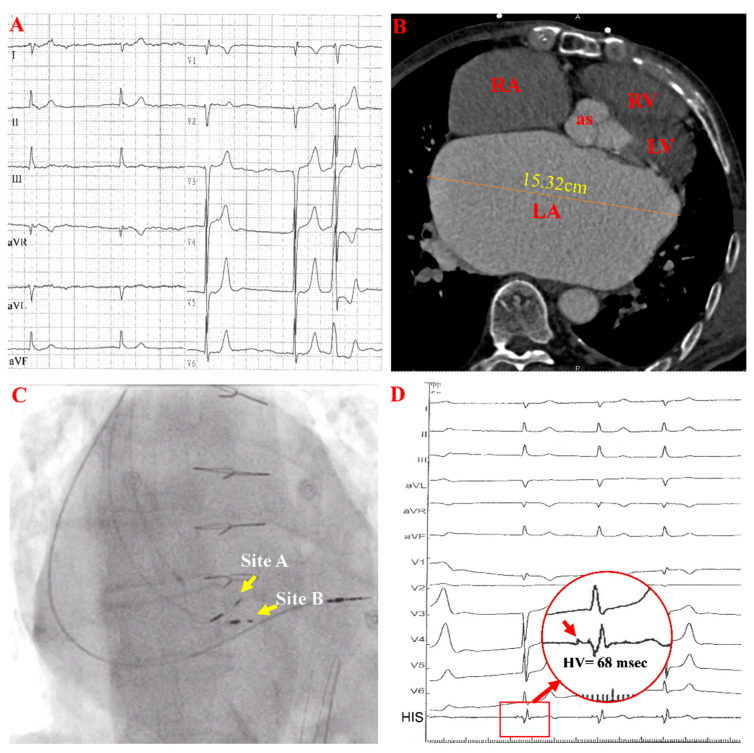
Images and ECGs before HBP. (**A**,**B**) show a preprocedural ECG and large left atrium in cardiac CTA. (**C**,**D**) are fluoroscopic AP views and electrograms with prolonged HV intervals during the procedure. In panel (**D**), the red arrow in the red circle indicates the HB potential. AP: anteroposterior projection; RA: right atrium; LA: left atrium; RV: right ventricle; LV: left ventricle; as: aortic sinus.

A 70-year-old man presented with exertional dyspnea and recurrent presyncope for two months and was admitted to our center. He had rheumatic heart disease and chronic AF for nearly 30 years without any sinus rhythm documented on multiple electrocardiograms (ECGs). Six years ago, he underwent mitral valve replacement (E100-29M-00, Epic, St. Jude Medical Inc., St. Paul, MN, USA) and tricuspid valvuloplasty for severe rheumatic mitral stenosis and tricuspid insufficiency. The admission ECG showed AF with a slow ventricular rate (Figure 1A). The 24 h ambulatory ECG demonstrated persistent AF with a slow ventricular rate of 46 beats per minute (BPM), and paroxysmal complete atrioventricular block (AVB) with a junctional escape rhythm. Coronary computed tomography angiography (CTA) revealed a severely enlarged left atrium (Figure 1B). A permanent pacemaker was indicated for symptomatic bradycardia. Although the left ventricular ejection fraction (LVEF) was preserved, the actual LVEF might have been overestimated due to mitral regurgitation. Moreover, considering that the patient was expected to have a ventricular pacing requirement most of the time and his apparent biatrial enlargement, we tried His-bundle pacing (HBP) to obtain better cardiac mechanical synchrony.

During implantation, HBP (Figure 1C, site A) using the Select Secure lead (model 3830, Medtronic Inc., Minneapolis, MN, USA) was attempted with right ventricular pacing (RVP) as a backup (Figure 1C). A clear His-bundle potential was observed, and the His-ventricular (HV) interval was 68 ms at site A (Figure 1D).

**Figure 2:**
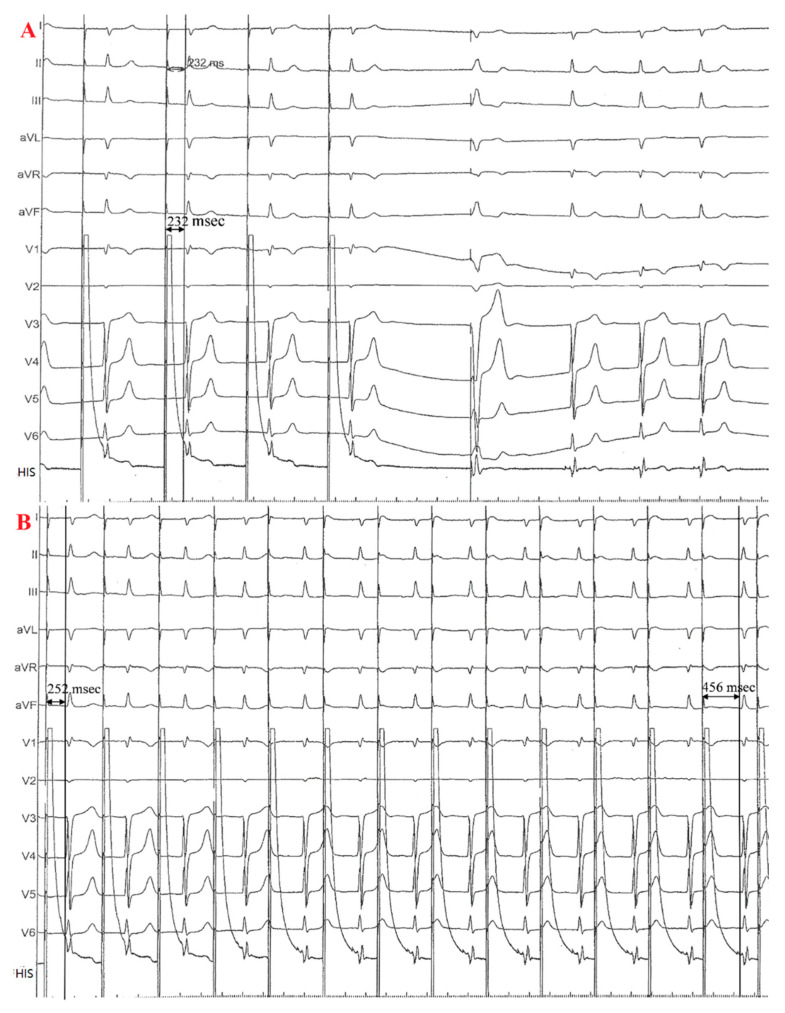
EGM during the implantation. (**A**), S-QRSonset was 232 ms when paced at a rate of 60 BPM at site A. (**B**), S-QRSonset progressively lengthened from 256 ms to 456 ms when the pacing rate was 90 BPM at site A.

The duration from the stimulus signal to the onset of the paced QRS complex (S-QRSonset) at site A was 232 ms when pacing at 60 BPM with a pacing threshold of 2.0 V/0.5 ms (Figure 2A). The S-QRSonset was longer than the baseline HV interval and showed progressive prolongation from 252 ms to 456 ms during pacing at a rate of 90 BPM (Figure 2B) with the same QRS morphology as the intrinsic conduction. This finding suggested that the pacing lead was at or close to the AVN.

**Figure 3:**
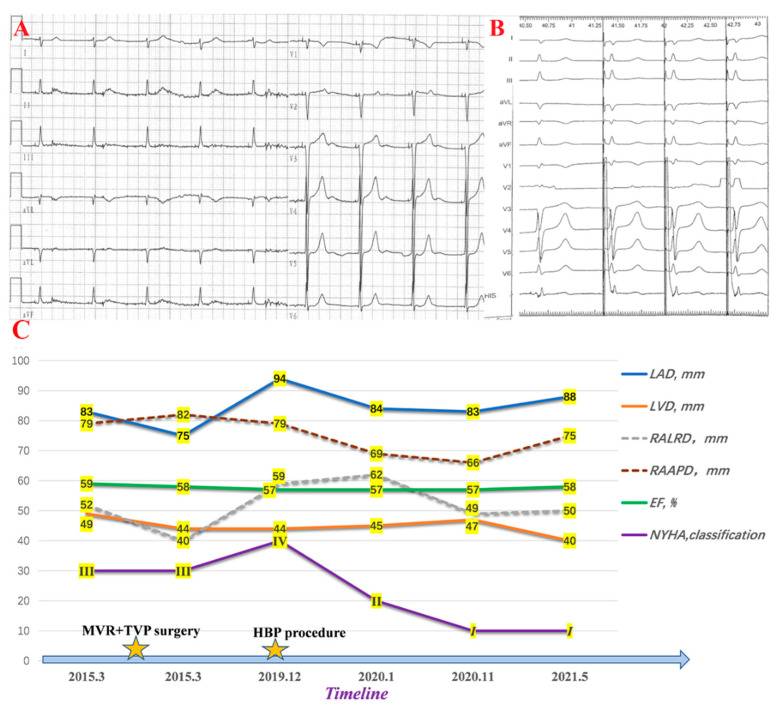
Changes after the HBP procedure. (**A**) shows postoperative ECG. (**B**) is the EGM of HBP. (**C**) shows changes in cardiac remodeling. LVD: left ventricular diameter; LAD: left atrial diameter; RALRD: right atrial left-right diameter; RAAPD: right atrial anteroposterior diameter; EF: ejection fraction; NYHA: New York Heart Association.

Then, we placed another 3830 lead distally at Site B with a final pacing threshold of 1.5 V/0.4 ms. The S-QRS interval at site B remained constant at 68 ms even if incremental pacing was performed, the same as the baseline HV interval (Figure 3A,B). Therefore, we chose site B, His-bundle pacing, as the final lead position to minimize progressive S-QRS prolongation.

The patient’s symptoms improved significantly at the 1.5 years follow-up with a stable and favorable pacing threshold and sense parameter, which the device program confirmed. Moreover, the sizes of both atria were reduced (Figure 3C).

**Figure 4:**
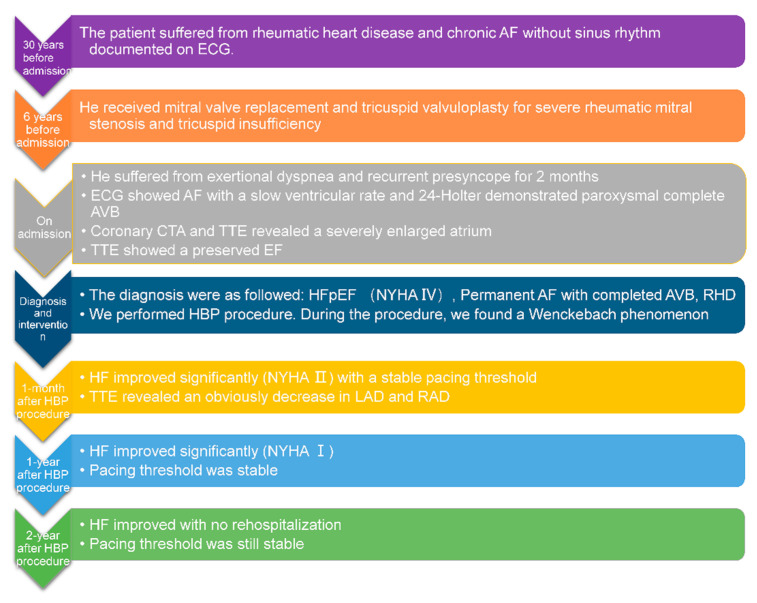
Timeline showing the clinical course in this patient.

The patient felt very good after the HBP procedure without symptoms of HF and was satisfied with the treatment. A timeline is showcased in Figure 4.

## 3. Discussion

This case would be the first report of intraoperative AVN pacing during AF, even if unintended. The atrioventricular conduction axis is a transition structure between atrial and ventricular components. It is mainly composed of the compact node, the penetrating bundle, the non-branching and branching atrioventricular bundle, and the right and left bundle branch [3]. The compact node, which is completely insulated and encircled by the fibrous tissue of the central fibrous body, becomes the penetrating bundle as the axis enters the atrioventricular component of the membranous septum. The WP is a well-known electrophysiological characteristic of the AVN. The conduction delay mainly occurred at the start of the penetrating bundle. The penetrating bundle’s position varies regarding its relation to the hinge of the septal leaflet of the tricuspid valve. AVN and penetrating bundle’s variable location probably facilitates AVN capture, especially when being before the hinge [2].

Pacemaker-related WP was once noted in a case of HB lead-induced current injury during sinus rhythm [4]. In that case, a prolongation of the PR interval and splitting of the His potential into two low-voltage components (H1-H2) were observed after the deployment of the pacing lead. Then, H1-H2 demonstrated a Wenckebach pattern with progressive prolongation of the H1-H2 interval until H1 dropped. In our case, no splitting of the His potential accompanied by the Wenckebach phenomenon was observed during the procedure, which might indicate no current injury. The HV interval stayed at 68 ms until the lead moved, reducing the likelihood that the observed decremental conduction was caused by lead-related trauma. Moreover, no atrial potential following the stimulus signal was recorded, and there might not be atrial capture since the patient had persistent AF. His bundle injury current was observed at site A on unipolar EGM [5]. The prolonged HV interval of 68 ms suggested impaired conduction system function. All clues may indicate that the pacing lead at site A might be at the distal end of the AVN because both the Wenckebach phenomenon and the HB potential were recorded. The lead happened to capture the AVN, indicating that it was not in the right atrium.

Of course, there are other possibilities to explain this phenomenon. We did not observe the splitting of His potential; however, the inability to record split potentials did not completely rule out the possibility of intra-Hisian conduction system disease. Additionally, there was probably atrial capture at site A with physiological Wenckebach AV block. As we can see delicate and discernible sawtooth waves on the surface ECG, we believe that atrial stand-steel should be ruled out.

AVN pacing may be possible and physiological, but it needs a high output and is more prone to the current injury. Therefore, considering the associated risks, including lower R-wave sense and higher threshold, we ultimately chose site B as the final lead position in the present case. One primary concern with HBP is the increased capture thresholds with time [6]. The mechanism for the delayed increase in the threshold remains unknown; it might be due to inadequate fixation, lead slack, local fibrosis, or disease progression. We chose site B and fixed the HB lead for better long-term pacing thresholds. In the present case, a stable and favorable pacing threshold (1.5 V/0.4 ms) and R wave amplitude (4.5 mV) were confirmed during the follow-up. However, whether these parameters will change in the coming years requires further follow-up.

Compared to traditional RVP, HBP can prevent ventricular desynchrony, optimize hemodynamics, and potentially correct bundle branch block. These advantages improve clinical outcomes and reduce arrhythmias, HF hospitalizations, and mortality [6,7]. This patient’s HF symptoms and atrial remodeling improved significantly during the two-year follow-up.

## 4. Conclusions

This case is the first reported case in which we achieved AVN capture in a patient with persistent AF. Although we ultimately chose HBP for better long-term pacing thresholds, the result of this case suggested that AVN pacing may be possible.

## Data Availability

All the data supporting our findings are contained within the manuscript.

## References

[1] Li J., Greener I.D., Inada S., Nikolski V.P., Yamamoto M., Hancox J.C., Zhang H., Billeter R., Efimov I.R., Dobrzynski H. (2008). Computer three-dimensional reconstruction of the atrioventricular node. Circ. Res..

[2] Cabrera J.-Á., Anderson R.H., Porta-Sánchez A., Macías Y., Cano Ó., Spicer D.E., Sánchez-Quintana D. (2021). The Atrioventricular Conduction Axis and its Implications for Permanent Pacing. Arrhythmia Electrophysiol. Rev..

[3] Macías Y., de Almeida M.C., Tretter J.T., Anderson R.H., Spicer D.E., Mohun T.J., Sánchez-Quintana D., Farré J., Back Sternick E. (2022). Miniseries 1—Part II: The comparative anatomy of the atrioventricular conduction axis. EP Eur..

[4] Anderson R.D., Prabhu M., Kalman J.M., Vohra J.K., Morton J.B., Stevenson I. (2019). Intra-Hisian Wenckebach Phenomenon during His Bundle Pacing: A Hazard Associated with “Precision” Medicine. JACC Clin. Electrophysiol..

[5] Jastrzębski M., Moskal P., Czarnecka D. (2019). Massive His bundle injury current corresponds with acute trauma and slowing of conduction that has to subside before pacing threshold assessment. J. Cardiovasc. Electrophysiol..

[6] Abdelrahman M., Subzposh F.A., Beer D., Durr B., Naperkowski A., Sun H., Oren J.W., Dandamudi G., Vijayaraman P. (2018). Clinical Outcomes of His Bundle Pacing Compared to Right Ventricular Pacing. J. Am. Coll. Cardiol..

[7] Sharma P.S., Vijayaraman P., Ellenbogen K.A. (2020). Permanent His bundle pacing: Shaping the future of physiological ventricular pacing. Nat. Rev. Cardiol..

